# Estimation of sexual dimorphism of adult human mandibles of South Indian origin using non-metric parameters and machine learning classification algorithms

**DOI:** 10.1038/s41598-025-17831-3

**Published:** 2025-10-03

**Authors:** John Valerian Corda, A. Karthikeyan, Mohammad Zuber, Meera Jacob, Amith Ramos, Mamatha Hosapatna, Anne Dsouza, Akhilesh Kumar Pandey, Vrinda Hari Ankolekar

**Affiliations:** 1 Department of Mechanical Engineering, Moodlakatte Institute of Technology, Kundapura, Karnataka India; 2 Department of Computer Science & Engineering (Data Science), Moodlakatte Institute of Technology, Kundapura, Karnataka India; 3 Department of Aeronautical & Automobile Engineering, Manipal Institute of Technology, Manipal Academy of Higher Education, Manipal, 576104 India; 4 Department of Anatomy, Yenepoya Medical College, Mangaluru, India; 5 Department of Anatomy & Medical Imaging, American University of Antigua College of Medicine, Antigua, Antigua and Barbuda; 6 Department of Anatomy, Kasturba Medical College, Manipal Academy of Higher Education, Manipal, 576104 India; 7 Department of Community Medicine, Kasturba Medical College, Manipal Academy of Higher Education, Manipal, 576104 India

**Keywords:** Mandible, Machine learning algorithms, Non-metric features, Sexual dimorphism, Synthetic minority oversampling technique, Random oversampling, Anatomy, Medical research, Engineering

## Abstract

The mandible is one of the most reliable in sex determination in forensic anthropology. The shape of the mandible provides valuable information regarding the male and female distinctions. Machine learning algorithms are widely used for various applications due to their accuracy and reliability, extending their application in biological profiling. This study aims to estimate sexual dimorphism using various machine-learning algorithms based on non-metric features of the mandible. This study uses four machine-learning algorithms—k-nearest neighbors, decision tree, support vector machines, and random forest to determine sex based on 12 mandibular non-metric parameters. The data was collected from three medical institutes in Karnataka, India, involving a sample of 156 individuals. Random Forest consistently achieved the highest Jaccard Index (0.86), F1 score (0.92), and accuracy (0.92) across both SMOTE and Random Over-Sampling (ROS) methods, showing stable and robust performance. ROS improved balanced accuracy for KNN, Decision Tree, and SVM by up to 9.7%. Feature importance analysis highlighted N6 Gonial angle and N12 Flexure ramal post border as key predictors. Statistical tests found no significant accuracy differences among models. Female specificity remained lower across all models. This study offers insights into employing machine learning algorithms for sex identification using non-metric observations of the mandible.

## Introduction

Sex determination using a mandible is vital due to its higher resistance to damage and disintegration. Studies have proved that the pelvis and mandible are most trustworthy in determining sex^1,2^. Due to its highly dimorphic characteristic, the mandible is one of the most reliable bones in the skull for sex determination^3^. A large database of literature supports this argument, which indicates the use of mandible bones for sex determination^4–7^.

In the events of mass disasters, the identity of the deceased is one of the important factors to consider. Forensic medicine and legal medical work revolve around sex identification as a significant element of the study. Research indicates that morphological features help identify sex with greater accuracy through complete skeletal analysis^8^. The unavailability of the complete skeleton poses difficulties in determining the sex in many cases^9^. Among all the human bones, the pelvis and skull are most dependable for sexual dimorphisms. The mandible is the skull’s most durable and strongest bone, and on unavailability of the pelvis, it becomes a principal source for sex confirmation^10^. The changes in age, sex, and race are directly related to the morphological features of the pelvis bone. Male and female mandibles are distinguished by general size, chin shape, gonial angle, and gonial flare^11^.

Non-metric or visual traits have emerged as a viable option for sex determination, as these traits can be evaluated swiftly and easily without the need for specialized equipment, thus rendering this approach particularly effective in field investigations^12^. They provide a feasible option for sex identification in the absence of metric characteristics, which may be unavailable due to fragmentation, trauma, or the unavailability of a reference sample. The advantage of non-metric evaluation is that it is conducted by visual observations. It is often organized according to an ordinal scoring system that encapsulates the spectrum of trait expression observed between male and female individuals, or it can be approached categorically. Non-metric traits can be employed without the complications of population affiliation, and consequently, they have become widely utilized in estimating biological sex^12,13^.

Machine Learning (ML) has become essential in biomedical applications. Biomedical models analyze data while identifying methods to improve systems, enhancing understanding of system goals, algorithms, and operational frameworks. ML leverages engineering applications for efficient data modeling without needing strong assumptions about the system. It offers a more precise data characterization than biomedical models, serving as a source of solutions and a key reference point. ML is a branch of artificial intelligence that employs statistical methods to improve systems’ ability to learn from data and make predictions or decisions. This enables algorithms to enhance their performance as they process more data over time. In biomedical engineering, ML algorithms can analyze complex datasets, identify patterns, and make informed decisions autonomously without human intervention^14^.

Integrating medicine and machine learning is inherently interdisciplinary, bringing together experts from various fields, including biology, medicine, engineering, and data science. This collaborative effort from different disciplines fosters the development of safe, ethical, and practical new tools, methodologies, and innovative inventions^15^. Machine learning significantly improves the efficiency and accuracy of healthcare delivery. It addresses challenges such as the shortage of medical professionals and the increasing demands on healthcare systems^16,17^. Recently, ML algorithms have been extensively used in many biomedical applications.

T. Wood et al.,^18^ predicted post-pubertal mandibular length in 163 males with Class I Angle malocclusion using machine learning algorithms, achieving an accuracy ranging from 95.80 to 97.64%. Similarly, M. T. A. Baban and D. N. Mohammad^19^ used Cone beam computed tomography (CBCT) radiograph images of 104 males and 104 females, converted into 3D models for volume, surface area, and 10 linear measurements. Among the machine learning algorithms employed for sex identification, Gaussian Naive Bayes performed best. Toneva et al.,^20^ analyzed cranial measurements of 169 males and 224 females of Bulgarian origin for sex determination using Support Vector Machines (SVM) and Artificial Neural Networks (ANN) algorithms, with SVM achieving 96.1% accuracy. Nogueira et al.,^21^ focused on radius bones from the French population, analyzing 16 measurements from the left radii of 36 males and 42 females, and achieved 97% accuracy with carefully selected variables. In a study by Knecht et al.,^22^, measurements from 180 skeletons and 21 forensic cases were analyzed using the patella’s height, breadth, and thickness for sex determination, where the SVM classifier achieved 91% accuracy. Guo et al.,^23^ examined linear measurements of the maxillary sinus (length, width, and height) from 477 samples of the Chinese Han population across all age groups. Using CBCT scans, the Random Forest method showed superior performance, with accuracy ranging from 77 to 88%. Curate et al.,^24^ investigated 15 femoral measurements for sex determination in 100 males and 100 females from the Portuguese population, reporting accuracy between 56.9% and 86.2% for univariable models and 84.5–89.7% for multivariable models. Lastly, Coelho J et al.,^25^ utilized pelvic bones for sex determination, analyzing 38 metric variables from a dataset of 256 individuals (131 females and 125 males). Their customized machine learning algorithm achieved 92% accuracy with three variables and 97% accuracy with all 38 variables. Published research has shown the use of Greylag Goose Optimization (GGO) method in cases where feature selection is required. An accuracy of 98.4% was achieved by using the Multilayer Perceptron (MLP) model for lung cancer classification^26^. A study on mechanical ventilation predictions using multilayer multitask Long Short-Term Memory (LSTM) models for classification and neural networks for regression, revealing higher scores in both accuracy and precision^27^. Cardiovascular disease detection using a novel framework using a snake optimization algorithm. This study, with a dataset of 14 attributes, emphasized the improvement of feature selection for ML models, and the model achieved a remarkable 99.9% in detecting cardiovascular disease. The algorithms used for the study were Random Forest, SVM, and XGBoost^28,29^. Recently, some studies have been conducted on sex determination using mandible data and seven ML algorithms, including support vector machines, random forest, among others; however, this study used data from CT scans of the adult population^30^. They found that the support vector machine algorithm achieved the highest accuracy of 95.3% and is well-suited for classification problems.

The literature review shows a notable scarcity of articles focused on applying classification machine learning algorithms, specifically utilizing mandibular datasets. This gap suggests that there is limited research exploring how these algorithms can be effectively employed to analyze and interpret data related to the mandible, which could provide valuable insights in fields such as sex identification. This study bridges the gap by applying various machine learning classification algorithms to nonmetric datasets of mandibles, which can be effectively used for sex detection. This study utilizes algorithms like decision trees (DT), support vector machines (SVM), k-nearest neighbors (KNN), and Random Forest (RF), which are discussed in detail in further sections.

## Materials & methods

### Overall methodology adopted for this work

**Fig. 1:**
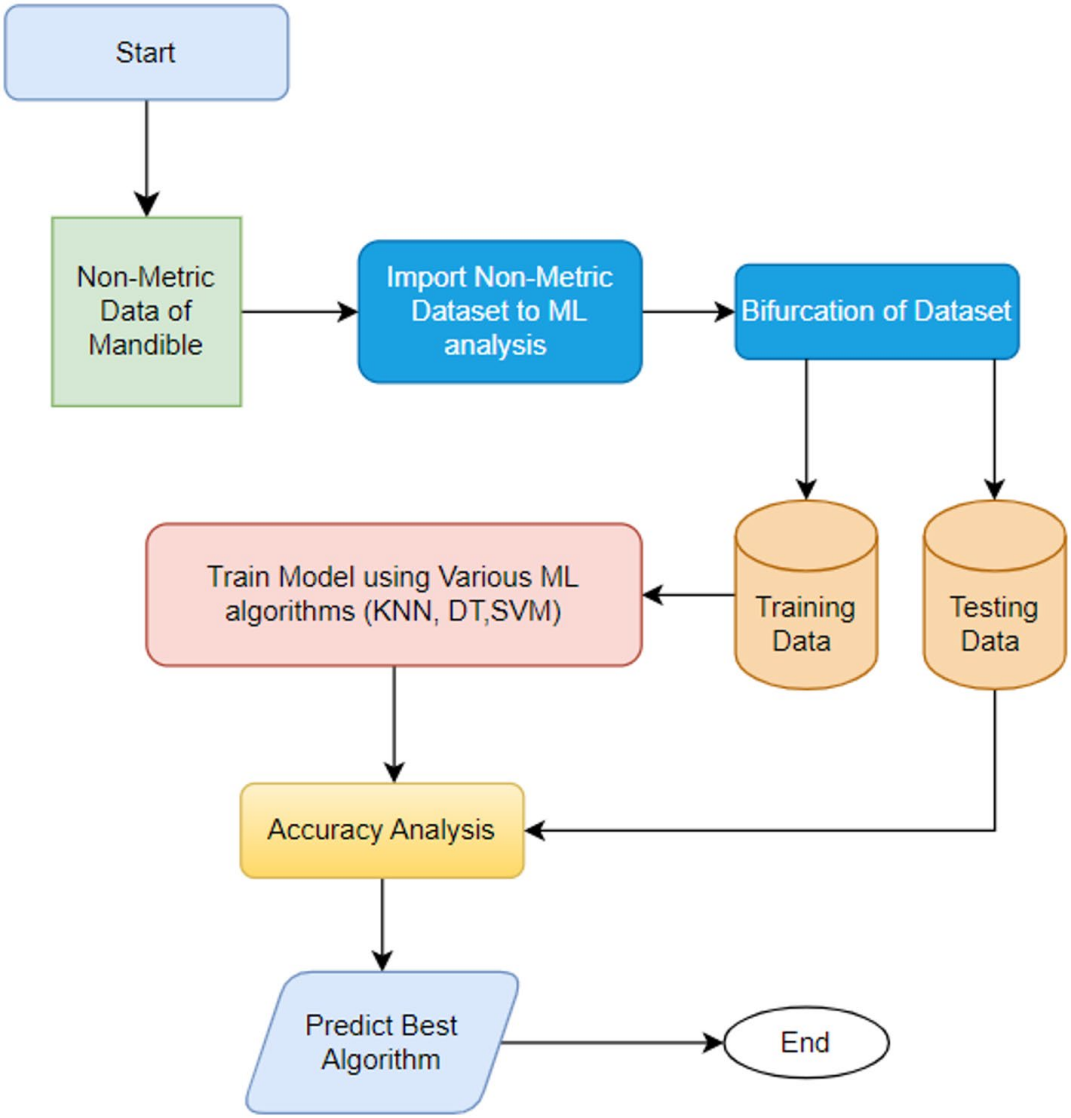
Overall Methodology.

This study encompasses significant contributions from two expansive and comprehensive fields, namely medicine and engineering, which are essential in furthering this research. Initially, the qualitative non-metric data concerning the various features of the mandibles is meticulously documented and systematically categorized. Moreover, the data that has been collected is utilized for comprehensive ML analysis, which constitutes a fundamental element in advancing the research objectives. The principal purpose of employing sophisticated ML algorithms is to optimize and refine the classification process of the mandibles, effectively categorizing them into male and female based on the specific non-metric data observed and recorded throughout the investigation. The data collection methodology and the machine learning classification algorithms used in this study are elaborated in detail in the subsequent sections. The overall framework of this work is shown in Fig. 1.

The dataset consists of 102 males and 54 females, indicating a significant class imbalance. This imbalance can lead to bias in machine learning models, ultimately reducing accuracy for the minority class. To mitigate this issue, we applied the SMOTE (Synthetic Minority Over-sampling Technique) and Random Over-sampling (ROS) methods to balance the classes. We have used one-hot encoding to convert categorical features into binary values. The non-metric mandibular features of this study did not possess any natural or ordinal structure. One-hot encoding ensured that the model treated each anatomical feature as independent to avoid misleading relational assumptions and enhanced feature interpretation.

### Data Collection – Non-metric parameters

This multicentre cross-sectional observational study was conducted on 156 adult human mandibles of known sex, sourced from three medical institutes in Karnataka, South India (102 male and 54 female). The mandibles were verified to be of South Indian origin based on records from each respective institute. Only adult mandibles with all components intact were included in the study, while pediatric and damaged mandibles were excluded. Approval for the study was obtained from the institutional ethics committee of the Kasturba Medical College, Manipal (IEC 476/2019), followed by written consent from the authorities of all the institutes involved in this study. Human Ethics and Consent to Participate declarations: Not applicable as our IEC waivered the individual consent.

The mandibles underwent non-metric observations.

We considered 12 nonmetric parameters based on the study by Hu et al.,^31^. We first numbered all the mandibles and observed for the parameter ‘N.’ To minimize the observer variability, five authors with equal experience and expertise in skeletal morphology observed each parameter independently. The final decision was based on the.

majority agreeing upon one trait/ parameter.

- N1: Shape of chin- Biolobate/square/pointed.
- N2: Profile of the chin- Vertical/prominent.
- N3: Lower border- Straight/rocker.
- N4: Ascending ramus shape- Pinched/wide.
- N5: Ascending ramus profile- Vertical/slanted/ inverted.
- N6: Gonial angle- Inverted/straight/ everted.
- N7: Inversion of post edge of ramus- Absent/slight/somewhat strong/strong.
- N8: Accessory mental foramen- Present/absent.
- N9: Mylohyoid bridge- Absent/partial/complete.
- N10: Retromolar foramen- Present/absent.
- N11: Accessory mandibular foramen- Absent/present.
- N12: Flexure of the Ramal posterior border- straight/flexure.

**Fig. 2:**
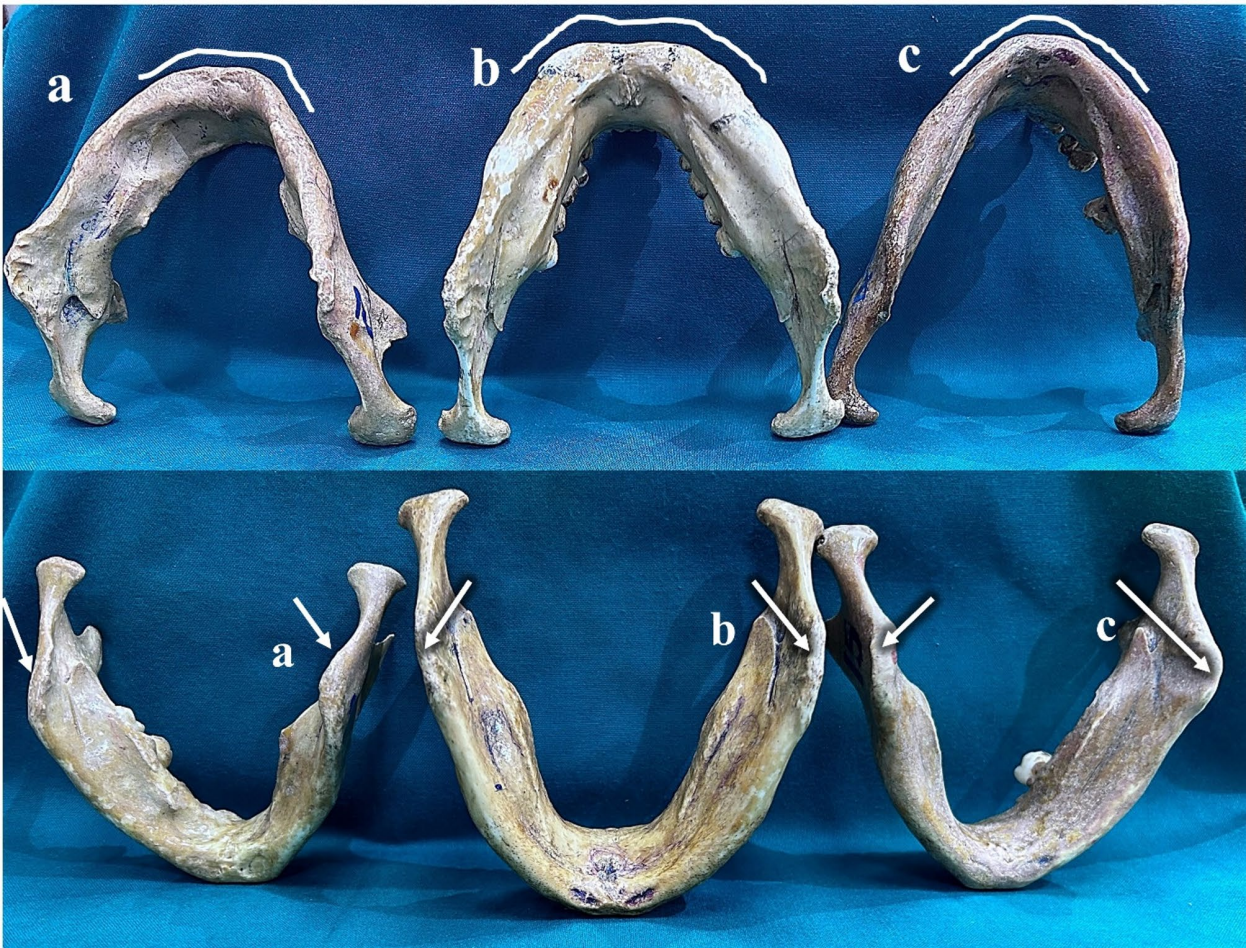
Inferior view of the mandibles depicting different chin shapes. (**a**)- Square, (**b**)- Bilobate, and (**c**)- Pointed and different profiles of the gonial angle. (**a**)- Inverted, (**b**)- Straight, and (**c**)- Everted.

**Fig. 3:**
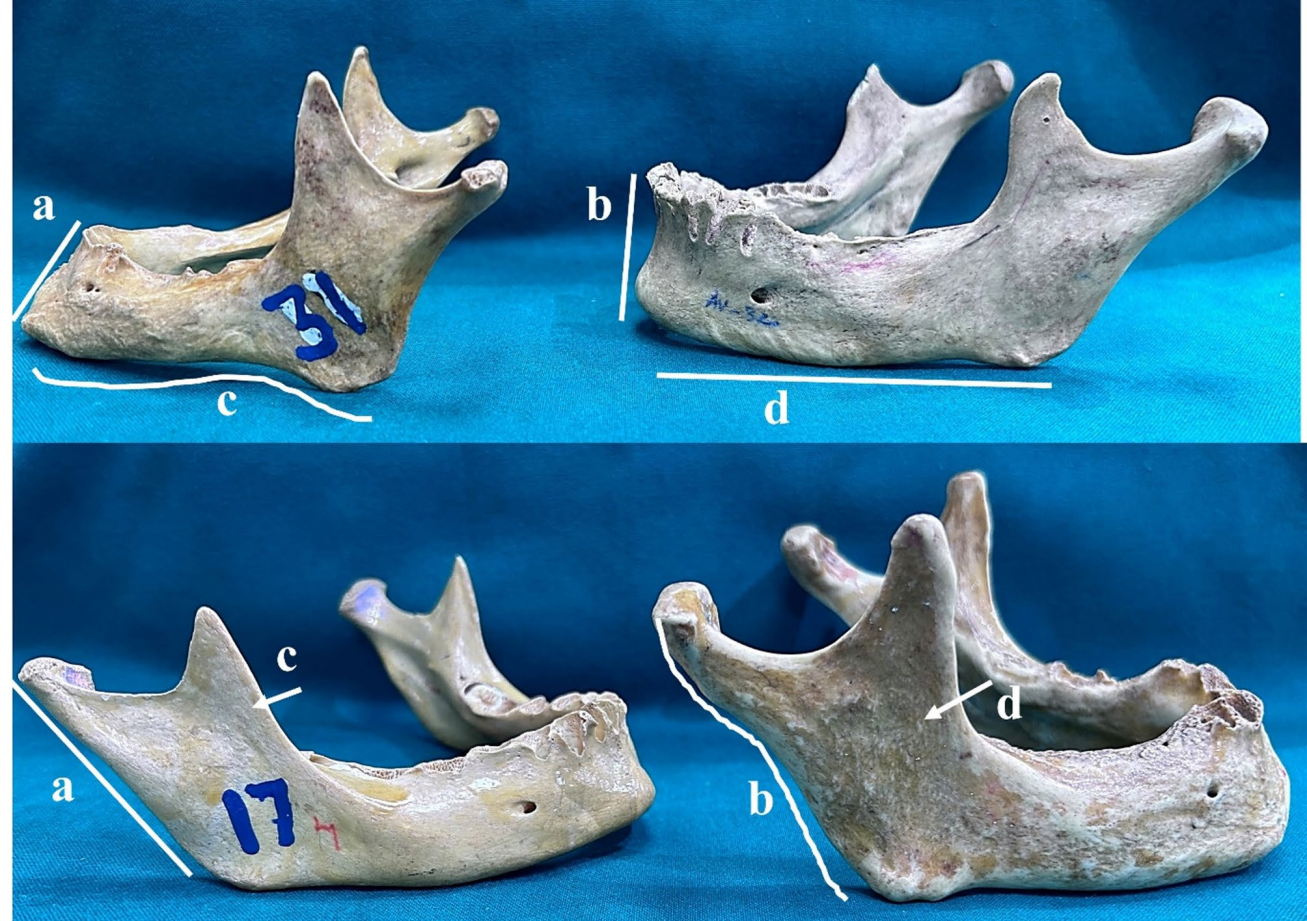
: Lateral view of the mandibles depicting different chin profiles. (**a**) Prominent and (**b**) Vertical. The lower border of the mandible showing (**c**) Rocker and (**d**) Straight profiles and different profiles of the ascending ramus. (**a**) Straight, (**b**) Flexure, (**c**) Wide, (**d**) Pinched.

Some of the variables observed for each parameter are displayed in Figs. 2 and 3.

### Machine learning models and statistical indicators

The KNN is one of the most commonly used ML classification algorithm. The majority class is obtained by a mechanism of voting in the KNN-based algorithm, where the most prevalent class among the KNN is designated. The user specifies the number of neighbors, n, to consider. This value is determined by identifying the n training instances with the smallest Euclidean distance in the input feature space. Equation 1 calculates the Euclidean distance, where x refers to the point under consideration, and y represents the nearest neighbor required to identify the closest neighbor.

1 $$d(x,y)=\sqrt {\sum\limits_{{i=1}}^{n} {{{\left( {yi - xi} \right)}^2}} } $$

The Decision Tree (DT) is a ML algorithm for tasks involving classifications. In this structure, internal nodes signify features, branches represent decision rules, and leaf nodes indicate outcomes. The top node, the root node, initiates decision-making by evaluating attribute values and recursively partitioning the tree. The tree is designed to make the classification decision based on the training datasets with class labels. As predictor variables are assessed, the navigation leads to either a left or right node, resulting in a final output class at the leaf node^32^.

Within every dataset, the partition is made using an entropy metric, measuring impurity or disorderliness in the dataset. Equation 2 represents the entropy, where n, S, and pi represent the data, unique classes, and a number of elements of class I, respectively.

2 $$Entropy(S)= - \sum\limits_{{i=1}}^{n} {{p_i}} {\log _2}\left( {{p_i}} \right)$$

The support vector machine (SVM) constitutes a supervised learning classification algorithm that analyzes data within feature space. This is helpful for categorizing the data points irrespective of whether they are linear or non-linearly separated. The most commonly used kernel functions in SVM are linear, polynomial, Radial Basis Function (RBF), and sigmoid. The similarity and diversity that is present in the sample sets is measured using a metric known as the Jaccard Index. It is defined as the ratio between the size of the intersection and the size of the union of two label sets. The data used in the training set are used to predict the actual labels in the testing case. This comparison helps assess the prediction model’s accuracy and identify improvement areas. Jaccard index is depicted by Eq. 3.

3 $$J(A,B)=\frac{{\left| {A \cap B} \right|}}{{\left| {A \cup B} \right|}}=\frac{{\left| {A \cap B} \right|}}{{\left| A \right|+\left| B \right| - \left| {A \cap B} \right|}}$$

The F1 score, as defined in Eq. 4, ranges from a maximum value of 1 to a minimum value of 0. It is the harmonic mean of precision and recall. The accuracy of the model to make positive predictions is known as precision, and information on the identification of positive instances is defined by the recall. The contribution of precision and recall for the F1 score is equal. By default, the F1 score is 0, which indicates that the true positives (TP), false negatives (FN), or false positives (FP) values are not present.

4 $$F1=\frac{{2*TP}}{{2*TP+FP+FN}}$$

The KNN model was optimized by manually adjusting the number of neighbors, testing values from 3 to 14. Additionally, a 10-fold cross-validation was performed, and the configuration that achieved the highest accuracy was selected. In the SVM model, GridSearchCV was utilized to evaluate various kernel functions, including ‘linear’, ‘rbf’, ‘poly’, and ‘sigmoid’. The optimal kernel function was determined based on the highest F1 macro score, employing 5-fold cross-validation. The Decision Tree code employs essential hyperparameter optimization through GridSearchCV, in combination with a 5-fold StratifiedKFold cross-validation strategy. It further fine-tunes key parameters like criterion, max-depth, min-samples-split, and min-samples-leaf using F1-macro to ensure balanced class performance. This method evaluates different parameter combinations to find the best model for generalizing to unseen data. For the RF model, hyperparameters were tuned using GridSearchCV with 5-fold stratified cross-validation to improve performance and handle class imbalance. The key parameters included n-estimators (number of trees), max-depth (tree depth), min-samples-split, and min-samples-leaf (which control the size and shape of the trees), and bootstrap (set to True for resampling).

In this research, the data for training and testing is divided into two parts, with 80% used for training and 20% for testing^33^. The random selection of the data confirms that the datasets that are selected represent the original datasets. Receiver Operating Characteristic (ROC) curves were generated using the ‘matplotlib.pyplot’ library for various predictive models. Each curve illustrates the balance between the true positive rate (sensitivity) and the false positive rate (1 - specificity) across a range of threshold values. The area under the curve (AUC) provides a quantitative assessment on the efficacy of the model for binary classification.

### Synthetic minority oversampling Technique(SMOTE) and random oversampling (ROS)

The dataset used in this study consists of 102 males and 54 females, which clearly signifies an imbalanced dataset, which implies that the number of samples in the majority class is more than those in the minority class. In this study, the 102 males represent the majority class, and the 54 females represent the minority class. It is imperative to note that the classification algorithms used in ML are inherently defined by assuming that the number of instances in each class is equal. To overcome the complexity of class imbalance, the Synthetic Minority Oversampling Technique, also known as SMOTE, is widely used in machine learning studies. SMOTE generates additional samples for the minority class synthetically, thereby resulting in an increased representation of the minority class in the dataset. Studies have shown that employing SMOTE increased the accuracy of classification algorithms, thereby providing a robust solution for handling imbalanced datasets^34,35^.

Random oversampling is another technique that is widely used in ML to address the problems involving class imbalance in the datasets. In random oversampling, the examples in the minority class are selected randomly and added to the training set, thereby randomly duplicating the examples in the minority class. Some of the published literature has shown that random oversampling works best for moderately imbalanced data^36^. However, there have also been contradicting results showing that the random oversampling or random undersampling methods do not significantly enhance the performance of the ML models^37^.

### Correlation and confusion matrix

The correlation matrix serves as a pivotal analytical instrument that elucidates the intricate relationships between pairs of numerical values. The values of correlation coefficients range between − 1 and + 1. The value of + 1 denotes correlation, which is perfectly positive, whereas − 1 denotes perfectly negative correlation. The value of 0 represents no correlation or no relationship between the variables.

The confusion matrix, commonly known as the error matrix, delivers a graphical depiction of the performance of an algorithm. In this matrix, the rows denote the predicted values, whereas the columns denote the actual values, and these roles can be interchanged.

It is comprised of four essential components: True Positive (TP), True Negative (TN), False Positive (FP), and False Negative (FN). True Positive (TP) has both actual and predicted values as positive. True Negative (TN) has both actual and predicted values as negative. False Positive (FP) has an actual value as negative, while the prediction is positive. False Negative (FN) has actual value as positive, while the prediction is negative.

Accuracy is the proportion of total correct predictions (both positive and negative) made by the model out of all predictions. It reflects the overall effectiveness of the model in classifying both classes correctly. However, in datasets with class imbalance, high accuracy may be misleading, as a model can perform well simply by predicting the majority class more often. Specificity measures the proportion of actual negative cases that are correctly identified by the model. It is particularly important in scenarios where avoiding false positives is critical. High specificity means the model rarely misclassifies negative samples as positive, ensuring reliability in identifying what the target is not. In combination with sensitivity (or recall), specificity helps evaluate the model’s performance across both classes in a balanced manner.

The efficacy of the confusion matrix can be evaluated through various metrics, including precision and recall, with the pertinent formulas detailed in established academic literature.

### Statistical analysis

The frequency of occurrence of each variable was calculated. Additionally, the occurrence of a variable between male and female mandibles was calculated using the Chi-square test. Intra and inter-observer variability were calculated using Cohen’s Kappa and Fleiss Kappa tests respectively. SPSS software, version 26, was used for statistical analysis (SPSS Inc, IBM, Chicago).

## Results

### Descriptive statistics

The frequency distribution of each variable for the parameters observed is provided in Table 1. Cohen’s kappa value of 0.81 to 1.00 was considered a very good strength of agreement for intra-observer variability. The Fleiss kappa test revealed k values ranging from 0.4 to 1.00 indicating moderate to very good strength of agreement in inter-observer variability. A few variables showed significant differences in their occurrence between male and female mandibles. The lower border of the mandible was predominantly rocker in males and straight in female mandibles (*p* < 0.001). The gonial angle was found to be everted in males (*p* < 0.001), followed by a straight profile. Partial and complete mylohyoid bridges were observed significantly more in male mandibles (*p* = 0.021). A flexure of the posterior border of the mandibular ramus was found to be a trait observed in male mandibles (*p* < 0.001). A detailed description of the occurrence of these variables between male and female mandibles is given in Table 2.

**Table 1:** Frequency distribution of all the variables observed.

S. No.	Parameter	Variable Observed	Frequency (*N* = 156)	Percentage
N1	Shape of chin	Bilobate	29	18.6
		Pointed	31	19.9
		Square	96	61.5
N2	Chin profile	Prominent	117	75
		Vertical	39	25
N3	Lower border	Rocker	86	55.1
		Straight	70	44.9
N4	Ascending Ramus shape	Pinched	64	41.0
		Wide	92	59
N5	Ascending Ramus profile	Inverted	13	8.3
		Slanted	82	52.6
		Vertical	61	39.1
N6	Gonial angle	Everted	71	45.5
		Inverted	39	25
		Straight	46	29.5
N7	Posterior Edge of ramus	Absent	92	59
		Slight	53	33.9
		Somewhat strong	9	5.8
		Strong	3	1.9
N8	Accessory mental foramen	Absent	136	87.2
		Present	20	12.8
N9	Mylohyoid bridge	Absent	75	48.1
		Complete	7	4.5
		Partial	74	47.4
N10	Retromolar foramen	Absent	131	84
		Present	25	16
N11	Accessory mandibular foramen	Absent	133	85.3
		Present	23	14.7
N12	Flexure ramal posterior border	Flexure	98	62.8
		Straight	58	37.2

**Table 2:** Comparison of the occurrence of variables between male and female mandibles.

S. No.	Parameter	Variable	Frequency in Male (*n* = 102)	Percentage within sex	Frequency in Female (*n* = 54)	Percentage within sex	Significance
N1	Shape of chin	Bilobate	19 (65.5%)	18.6%	10 (34.5%)	18.5%	0.618
		Pointed	18 (58.1%)	17.6%	13 (41.9%)	24.1%	
		Square	65 (67.7%)	63.7%	31 (32.3%)	57.4%	
N2	Chin profile	Prominent	76 (65%)	74.5%	41 (35%)	75.9%	0.84
		Vertical	26 (66.7%)	25.5%	13 (33.3%)	24.1%	
N3	Lower border	Rocker	72 (83.7%)	70.6%	14 (16.3%)	25.9%	< 0.001*
		Straight	30 (42.9%)	29.4%	40 (57.1%)	74.1%	
N4	Ascending ramus shape	Pinched	42 (65.6%)	41.2%	22 (34.4%)	40.7%	1.000
		Wide	60 (65.2%)	58.8%	32 (34.8%)	59.3%	
N5	Ascending ramus profile	Inverted	11 (84.6%)	10.8%	2 (15.4%)	3.7%	0.287
		Slanted	51 (62.2%)	50%	31 (37.8%)	57.4%	
		Vertical	40 (65.6%)	39.2%	21 (34.4%)	38.9%	
N6	Gonial angle	Everted	63 (88.7%)	61.8%	8 (11.3%)	14.8%	< 0.001*
		Inverted	14 (35.9%)	13.7%	25 (64.1%)	46.3%	
		Straight	25 (54.3%)	24.5%	21 (45.7%)	38.9%	
N7	Inversion of the posterior edge of the ramus	Absent	62 (67.4%)	60.8%	30 (32.6%)	55.6%	0.459**
		Slight	32 (61.5%)	31.4%	20 (38.5%)	37%	
		Somewhat strong	7 (77.8%)	6.9%	2 (22.2%)	3.7%	
		Strong	1 (33.3%)	1%	2 (66.7%)	3.7%	
N8	Accessory mental foramen	Absent	87 (64%)	85.3%	49 (36%)	90.7%	0.452
		Present	15 (75%)	14.7%	5 (25%)	9.3%	
N9	Mylohyoid bridge	Absent	43 (57.3%)	42.2%	32 (42.7%)	59.3%	0.021*
		Complete	7 (100%)	6.9%	0	0	
		Partial	52 (71.2%)	51%	21 (28.8%)	38.9%	
N10	Retromolar foramen	Absent	85 (64.9%)	83.3%	46 (35.1%)	85.2%	0.823
		Present	17 (68%)	16.7%	8 (32%)	14.8%	
N11	Accessory mandibular foramen	Absent	85 (63.9%)	83.3%	48 (36.1%)	88.9%	0.478
		Present	17 (73.9%)	16.7%	6 (26.1%)	11.1%	
N12	Flexure of the posterior ramus	Flexure	78 (79.6%)	76.5%)	20 (20.4%)	37%	< 0.001*
		Straight	24 (41.4%)	23.5%	34 (58.6%)	63%	

* As calculated by the Chi-square test. P<0.05 is considered significant.

**As calculated by the Fisher Exact test.

### Correlation and confusion matrix

**Fig. 4:**
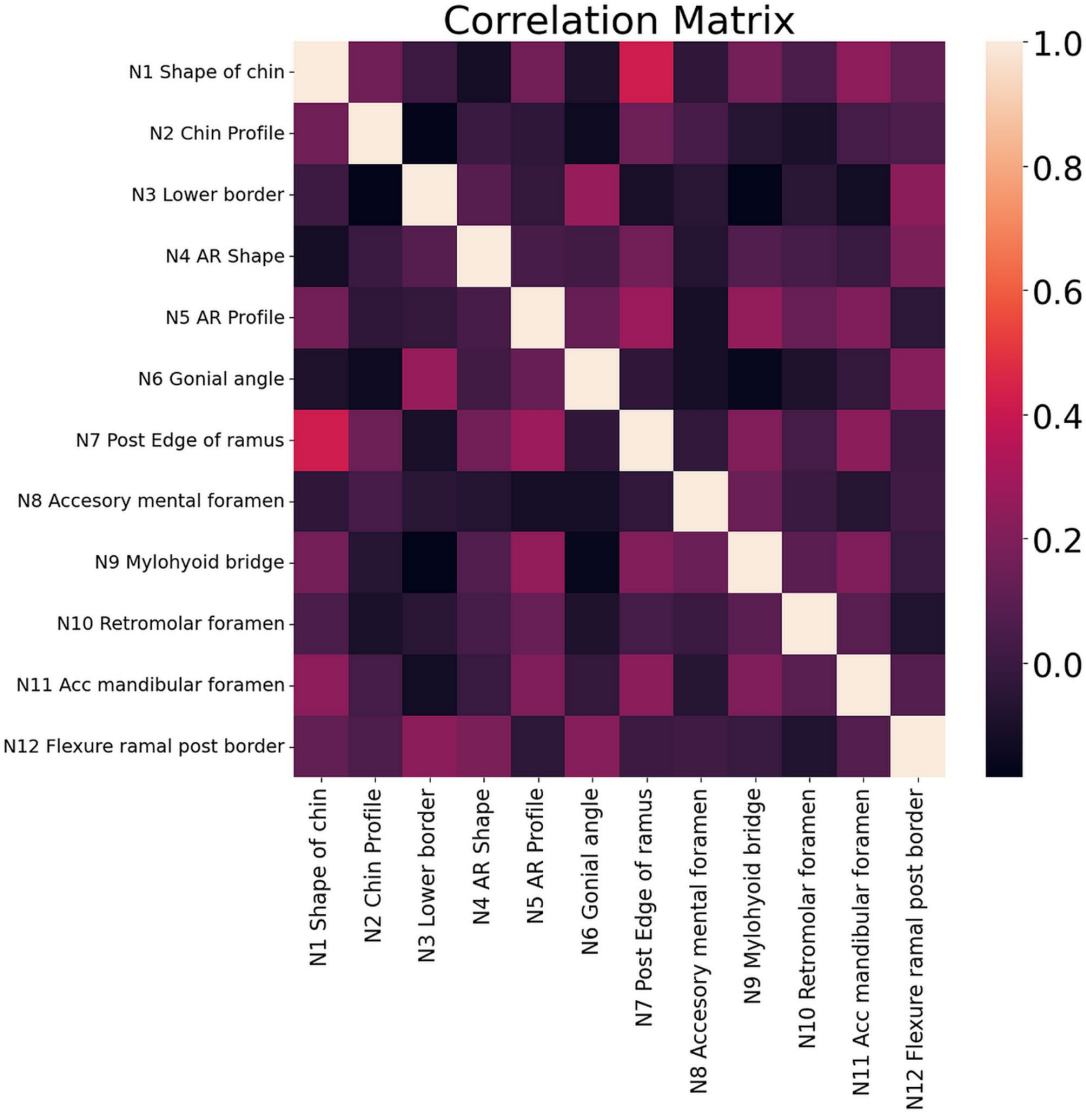
Correlation matrix.

The correlation between the variables used in this study is shown in Fig. 4 which is also called the correlation matrix. The correlation values typically range from + 1 to -1, where + 1 indicates a perfect positive correlation, 0 indicates no correlation, and − 1 indicates a perfect negative correlation between the variables.

**Fig. 5:**
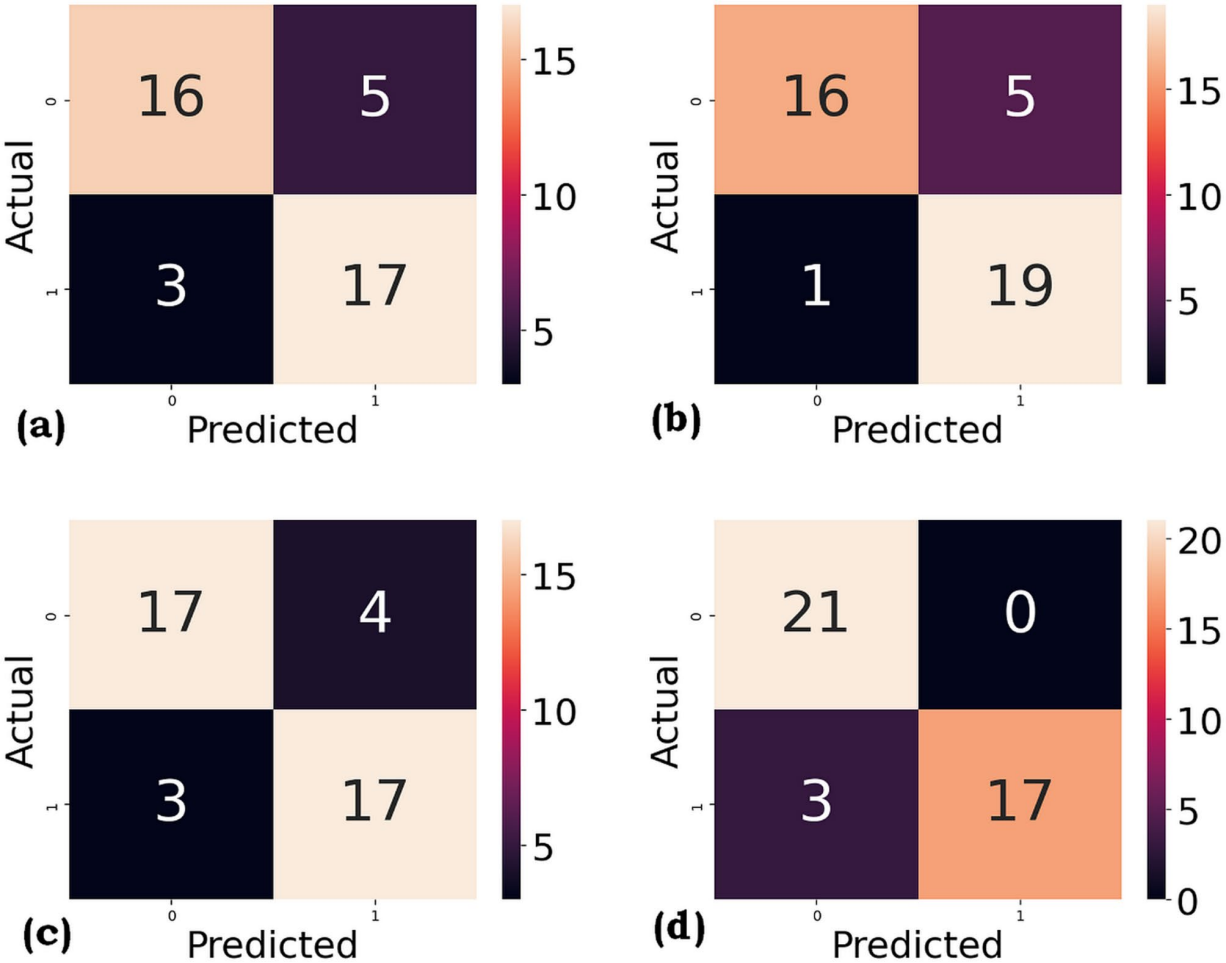
Confusion matrix (**a**) KNN (**b**) DT (**c**) SVM (**d**) RF for SMOTE.

**Fig. 6:**
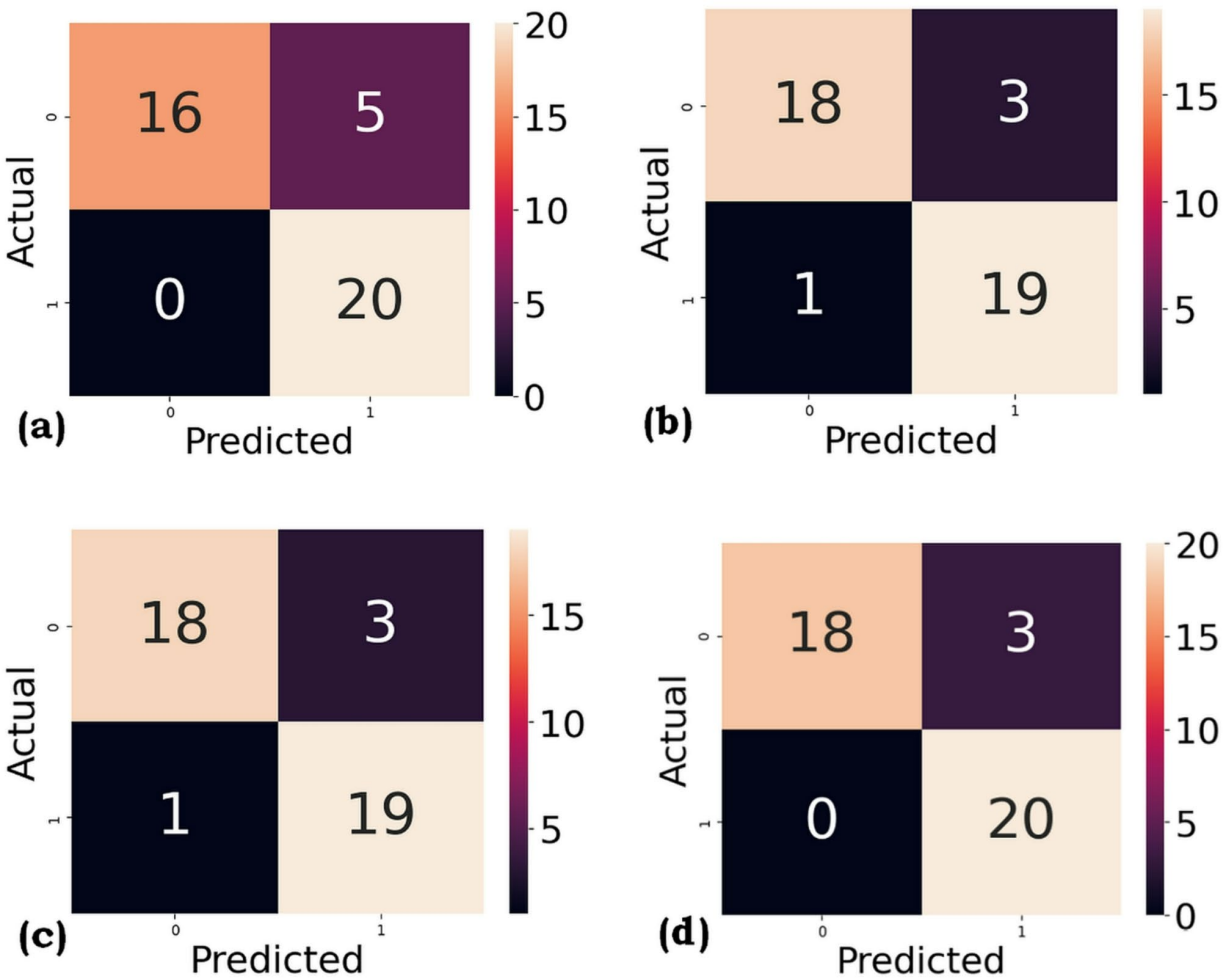
(**a**) KNN (**b**) DT (**c**) SVM (**d**) RF for ROS.

The confusion matrix for the four ML algorithms used in this work is shown in Figs. 5 and 6, respectively, for SMOTE and ROS.

The comparison of classification models using weighted average precision and recall provides valuable insights into both the accuracy and reliability of each model. RF stands out with the highest weighted average precision of 0.94 and recall of 0.93 for SMOTE and ROS, indicating that it not only makes highly accurate positive predictions but also captures the majority of true positives across all classes. This strong performance underscores RF’s capability to balance precision and completeness in predictions, making it highly suitable for applications that require both high accuracy and thorough identification. SVM and DT both achieved a weighted average precision and recall of 0.87 and 0.85 for SMOTE and average precision and recall of 0.91 and 0.90 for ROS, suggesting that they are similarly effective in balancing false positives and false negatives. These models offer a strong balance among interpretability, computational efficiency, and predictive performance. Although they do not reach the performance level of RF, they still represent a good trade-off, making them suitable options when model simplicity or speed is prioritized. On the other hand, KNN recorded the lowest weighted precision and recall of 0.81 and 0.80 for SMOTE and 0.91 and 0.90 for ROS. Although the differences may seem marginal, they reflect that KNN is less capable of accurately identifying all classes, especially in scenarios with overlapping class boundaries or imbalanced distributions. This lower performance suggests KNN may be more affected by local sample noise and might benefit from feature scaling or dimensionality reduction.

Overall, the RF model stands out as the most balanced and effective in terms of both precision and recall, followed by SVM and DT. The KNN model falls slightly behind in performance. These results highlight the effectiveness of ensemble methods in achieving robust and reliable classification, especially when dealing with real-world datasets that feature varied and complex patterns.

### Jaccard score, F1 score, accuracy, and specificity

The Jaccard index constitutes a significant metric utilized for assessing the similarity between predicted and actual classifications. An elevated Jaccard index indisputably indicates enhanced model efficacy and highly precise predictions. The F1 score, which embodies the harmonic mean of precision and recall, is crucial for appraising model performance, particularly in scenarios characterized by imbalanced class distributions. An F1 score approaching 1 illustrates a commendable equilibrium between precision and recall, distinctly reflecting the model’s strong performance and dependability. The Table 3 presents the consolidated Jaccard Index and F1 scores for the four ML algorithms used in this study.

**Table 3:** Summary of Jaccard index and F1 scores.

Algorithm	SMOTE		Random oversampling	
	Jaccard index	F1 score	Jaccard index	F1 Score
KNN	0.67	0.80	0.78	0.87
DT	0.74	0.85	0.82	0.90
SVM	0.70	0.82	0.82	0.90
RF	0.86	0.92	0.86	0.92

It can be seen from Table 3 that the KNN algorithm exhibits the least effective performance among the evaluated models, with Jaccard and F1 scores recorded at 0.67 and 0.80, respectively, for SMOTE and Jaccard and F1 scores of 0.78 and 0.87, respectively, for ROS. This indicates that KNN struggles with noisy features, as it is particularly sensitive to local distributions and has difficulty generalizing effectively in scenarios characterized by imbalanced or high-dimensional datasets. The DT demonstrates superior performance in comparison to KNN, achieving Jaccard and F1 scores of 0.74 and 0.85, respectively, for SMOTE and Jaccard and F1 scores of 0.82 and 0.90, respectively, for ROS. This finding shows that DT establishes a more systematic and rule-based decision boundary by executing hierarchical splits grounded in feature thresholds. Such a structured methodology facilitates enhanced generalization capabilities for the DT, especially in instances where the dataset exhibits non-linear separability. The SVM achieves a Jaccard score that is similar to that of the decision tree (DT); however, its F1 score is slightly lower. This suggests that while the SVM may demonstrate higher precision, it also has lower recall, meaning it is able to accurately identify fewer true positive cases. The RF model distinctly surpasses all other models, attaining the highest Jaccard index, which signifies a superior degree of prediction overlap with the true labels. Additionally, it showcases the most favorable F1 score, indicative of an optimal equilibrium between precision and recall. This remarkable performance can be ascribed to the ensemble nature of RF, which mitigates the risk of overfitting, adeptly captures intricate feature interactions, and generalizes effectively across diverse subsets of the dataset.

The Tables 4 and 5 represent the accuracy and class-wise specificity for all four models used in this study, based on results obtained after applying SMOTE and ROS techniques to the original dataset.

**Table 4:** Accuracy.

Algorithm	SMOTE		ROS	
	Accuracy	Confidence interval	Accuracy	Confidence interval
KNN	0.80	[0.75, 0.97]	0.87	[0.78, 0.97]
DT	0.85	[0.73, 0.95]	0.90	[0.80, 0.97]
SVM	0.82	[0.70, 0.92]	0.90	[0.80, 0.97]
RF	0.92	[0.80, 0.97]	0.92	[0.82, 1.00]

From Table 4 it is observed that for KNN, the accuracy was increased from 0.80 using SMOTE to 0.87 using ROC. The confidence intervals for accuracy of KNN were about [0.75,0.97], which suggests that there is 95% confidence that the model’s actual accuracy falls between 75% and 97%. The accuracy of DT and SVM increased from about 0.82 or 82% for SMOTE to 90% for ROS with a confidence interval of [0.70,0.92] for SMOTE and [0.80,0.97] for ROS. However, the RF algorithm exhibited the highest accuracy for SMOTE and ROS when compared to other algorithms. The accuracy for SMOTE is notably 8–15% greater for RF in comparison to KNN, DT, and SVM. It is also worth noting that the accuracy of RF for both SMOTE and ROS was the same.

The superior performance of RF can be attributed to its ensemble structure, which effectively reduces overfitting, captures complex feature interactions, and ensures better generalization across different data folds.

**Table 5:** Specificity per class.

Algorithm	Specificity per class (SMOTE)		Specificity per class (ROS)	
	Male	Female	Male	Female
KNN	0.76	0.85	0.76	1.00
DT	0.76	0.95	0.86	0.95
SVM	0.81	0.85	0.86	0.95
RF	0.86	1.00	0.86	1.00

A specificity value of 0.76 for Males and 0.85 for Females was achieved for KNN, which shows a better performance in correctly rejecting Male instances when evaluating Female samples.

Male specificity remains at 0.76 for ROS, while Female specificity improves to a perfect 1.00, showing enhanced ability to identify Female negatives. Decision Tree (DT) under SMOTE records equal specificity for Males (0.76) and high specificity for Females (0.95). ROS notably increases Male specificity to 0.86, while Female specificity stays stable at 0.95, suggesting improved precision in rejecting Male negatives. SVM resulted in a moderate specificity with SMOTE, which is 0.81 for Males and 0.85 for Females. However, the specificity improves to 0.86 and 0.95, respectively, with ROS, indicating better class distinction. Random Forest (RF) achieves the highest baseline specificity with SMOTE, which is 0.86 for Males and perfect 1.00 for Females. Notably, these values remain consistent with ROS, indicating robustness across oversampling methods in accurately identifying negatives for both classes.

Both SMOTE and ROS effectively mitigate class imbalance and enhance model specificity; however, ROS frequently produces superior or equivalent specificity metrics, particularly for the Male class across various algorithms. The Random Forest algorithm exhibits the most robust overall performance, demonstrating resilience to the oversampling method, achieving near-optimal specificity for both classes. This finding suggests that Random Forest is a dependable option for classification tasks involving this imbalanced dataset, with ROS potentially augmenting performance in other modelling scenarios.

McNemar’s test statistic evaluation is a non-parametric statistical examination employed to assess variations in paired nominal data, especially in the context of comparing the proportions of two interrelated groups. We have used McNemar’s test to evaluate if there is a significant difference in accuracy between two paired classification algorithms, which are shown in Table 6 for SMOTE and Table 7 for ROC. The p-value indicates the likelihood that observed differences between two classifiers are due to chance, with values below 0.05 suggesting significance. McNemar’s test statistic measures the frequency of disagreements in predictions and follows a chi-square distribution; a higher value indicates a greater difference. Together, they help determine if the classifiers significantly differ in accuracy.

**Table 6:** McNemar’s test for accuracy with SMOTE.

Algorithms compared		McNemar’s test statistic value	*p*-value	Inference
KNN	SVM	2	1	No significant difference in accuracy
KNN	DT	3	0.72	
KNN	RF	2	0.17	
DT	SVM	4	1	
DT	RF	3	0.50	
SVM	RF	1	0.21	

**Table 7:** McNemar’s test for accuracy with ROC.

Algorithms Compared		McNemar’s test statistic Value	*p*-value	Inference
KNN	SVM	1	1	No significant difference in accuracy
KNN	DT	2	1	
KNN	RF	0	0.5	
DT	SVM	1	1	
DT	RF	1	1	
SVM	RF	0	1	

It can be inferred from Tables 6 and 7 that the p-values exceed typical significance levels (e.g., 0.05), which indicates that there is no statistically significant difference in accuracy between the compared algorithms. All pairs of algorithms tested perform similarly in terms of accuracy when using SMOTE, and none of the differences are statistically significant according to McNemar’s test.

### ROC curve and AUC

**Fig. 7:**
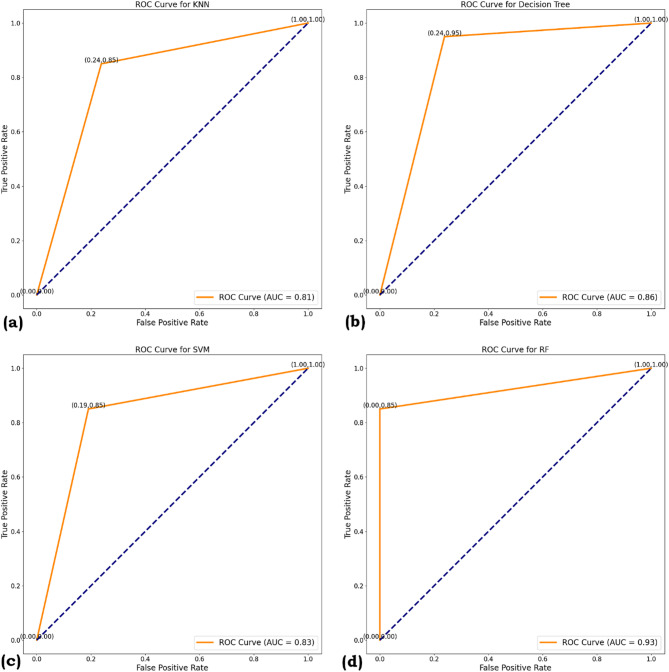
ROC Curve (**a**) KNN (**b**) DT (**c**) SVM (**d**) RF for SMOTE.

**Fig. 8:**
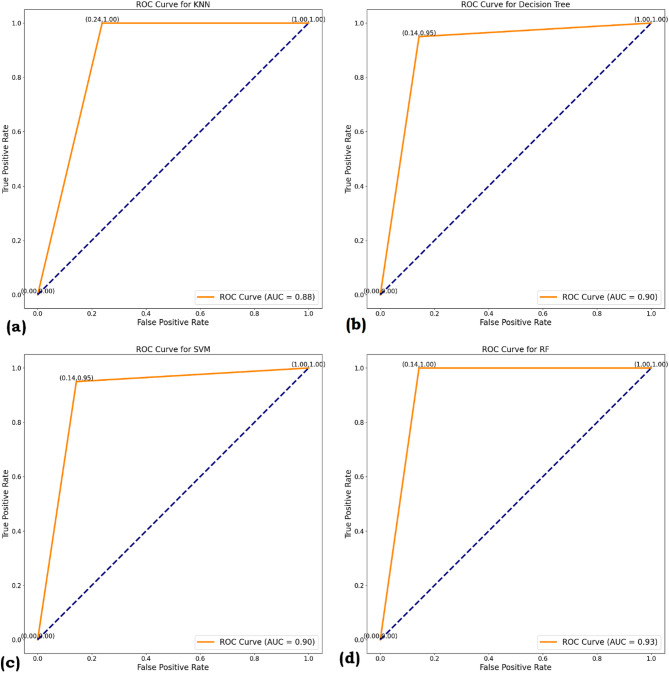
ROC Curve (**a**) KNN (**b**) DT (**c**) SVM (**d**) RF for ROS.

ROC (Receiver Operating Characteristic) and AUC (Area Under the Curve) are essential metrics for evaluating the performance of binary classification problems. A model provides better class estimates when the ROC curve is closer to the top left corner. A single number indicates the overall performance of the ROC curve, which is inferred by the AUC, the value of which lies between 1 and 0. The Figs. 7 and 8 summarizes the ROC and AUC for this work for SMOTE and ROS, respectively.

The Area Under the Curve (AUC) scores provide a measure of each model’s ability to distinguish between classes across all threshold settings. It can be noted from Fig. 7 RF achieved the highest AUC of 0.83, indicating strong discriminatory power and reliable performance across different classification thresholds. DT and SVM, followed by AUC scores of 0.86 and 0.83, respectively, showing moderate effectiveness. KNN recorded the lowest AUC of 0.81, suggesting it is less capable of consistently separating the classes. Figure 8 shows all four models show strong classification performance, with AUC values over 0.85. Random Forest (RF) leads at 0.93, indicating superior accuracy and fewer false positives. DT and SVM each score 0.90, while KNN has a slightly lower AUC of 0.88. The ROC curves visually support these results, with RF, DT, and SVM clearly outperforming KNN. These results highlight Random Forest as the most robust model in terms of overall class separation, while KNN may struggle to handle overlapping class distributions or more complex decision boundaries.

Bootstrap AUROC Difference is a statistical method used for the comparison of the Area Under the Receiver Operating Characteristic Curve (AUROC or AUC) between two classification models by estimating the variability of their difference. In this process, the sampling of the datasets with replacements is done, resulting in bootstrapped samples. For each of these samples, the difference in AUROC is calculated and recorded. The estimation of variability and confidence intervals is then evaluated based on the distribution of AUROC differences. By analysing this distribution, it is possible to determine if the performance difference is statistically significant or due to chance. The Tables 8 and 9 show the AUROC difference with SMOTE and ROS, respectively.

**Table 8:** AUROC difference with SMOTE.

Algorithms compared		AUROC difference	95% CI	Inference
KNN	SVM	-0.01	[-0.18, 0.13]	No significant difference in AUROC between the algorithms
KNN	DT	0.12	[-0.03, 0.30]	
KNN	RF	0.07	[-0.02, 0.18]	
DT	SVM	-0.15	[-0.40, 0.06]	
DT	RF	-0.05	[-0.21, 0.11]	
SVM	RF	0.09	[-0.02, 0.24]	

**Table 9:** AUROC difference with ROC.

Algorithms compared		AUROC difference	95% CI	Inference
KNN	SVM	0.01	[-0.11, 0.13]	No significant difference in AUROC between the algorithms
KNN	DT	0.05	[-0.11, 0.26]	
KNN	RF	0.06	[-0.08, 0.22]	
DT	SVM	-0.04	[-0.24, 0.13]	
DT	RF	0.001	[-0.15, 0.14]	
SVM	RF	0.05	[-0.05, 0.17]	

Both tables compare pairs of algorithms using the bootstrap method to estimate differences in Area Under the Receiver Operating Characteristic (AUROC), along with 95% confidence intervals (CIs). In Table 8, which includes Synthetic Minority Over-sampling Technique (SMOTE), the AUROC differences between models range from − 0.15 to 0.12, with all confidence intervals including zero. Similarly, Table 9, which presents ROC data, shows differences ranging from − 0.04 to 0.06, with all confidence intervals also containing zero. Since zero falls within every confidence interval, we can conclude that there is no significant difference in AUROC between any pairs of compared algorithms under both conditions. This indicates that all models perform comparably regarding their classification accuracy.

### Balanced accuracy and Matthews correlation coefficient (MCC)

When the dataset is imbalanced, balanced accuracy is the metric that is most widely used to evaluate the classification models. Balanced accuracy averages the recall scores of all classes, giving equal importance to each. For binary classification, it is the mean of the true positive rate (sensitivity) and true negative rate (specificity), ensuring fair assessment for both majority and minority classes. Balanced accuracy for the ML algorithms used in this work are presented in Table 10.

**Table 10:** Balanced accuracy.

Algorithm	Balanced accuracy(SMOTE)	Balanced accuracy (ROS)
KNN	0.80	0.88
DT	0.82	0.90
SVM	0.85	0.90
RF	0.92	0.92

As can be seen from Table 10 that there is an increase in the values of balanced accuracy across all the ML algorithms in the ROS method of class balancing. KNN showed an increase of 2.5%, whereas DT and SVM showed an increase of 9.7% and 5.8% respectively, for ROS when compared to SMOTE. Contrastingly, RF showed no increase in balanced accuracy across both the oversampling methods. This finding demonstrates its robustness to the choice of oversampling method. Overall, ROS seems to offer a slight advantage for most models, except for Random Forest, which performs equally well with both techniques.

The Matthews Correlation Coefficient (MCC) is a metric used to assess the performance of binary (two-class) classifications. When the dataset is imbalanced, MCC offers a balanced metric for accuracy evaluation. The Matthews Correlation Coefficient for the ML algorithms used in this work is presented in Table 11.

Table 11 shows that ROS generally increases the MCC values when compared to SMOTE across all the ML algorithms used in this work. KNN improves from 0.61 with SMOTE to 0.78 with Random Over Sampling (ROS), while SVM rises from 0.65 to 0.80. Decision Trees (DT) increase from 0.72 to 0.80 with ROS. Random Forest (RF) achieves the highest Matthews Correlation Coefficient (MCC) of 0.86, showing consistent performance with both SMOTE and ROS. Overall, ROS enhances the correlation between predicted and actual outcomes for most algorithms, with Random Forest consistently performing well.

**Table 11:** Matthews correlation coefficient (MCC).

Algorithm	Matthews correlation coefficient (SMOTE)	Matthews correlation coefficient (ROS)
KNN	0.61	0.78
DT	0.72	0.80
SVM	0.65	0.80
RF	0.86	0.86

### Permutation feature importance and Gini index

**Fig. 9:**
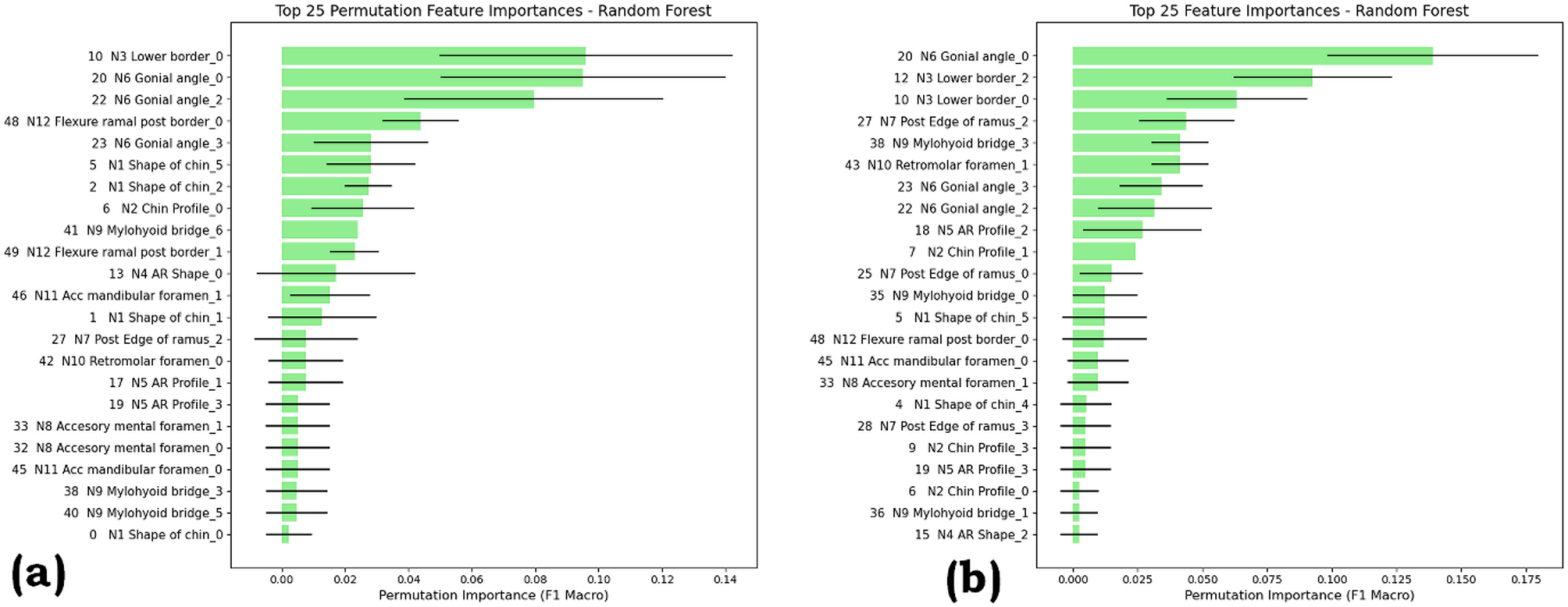
Permutation feature importance plot for the random forest model with (**a**) SMOTE (**b**) ROS.

The permutation feature importance for the Random Forest Model is as shown in Fig. 9 for SMOTE and ROS. The most critical features for SMOTE are the N3 Lower border_0, N6 AR profile_2, and N6 Gonial angle_2. They have the importance scores ranging between 0.09 and 0.12. Other features like N12 Flexure ramal post border_0 and N1 Shape of chin_0, also have a higher value of importance scores, suggesting that both angular and shape-based measurements are important for classification. The distribution of importance scores reveals that a few features have a strong influence, while many others contribute moderately, highlighting SMOTE’s ability to maintain variability across predictors.

For the ROS technique, N6 Gonial angle_0, N12 Lower border_2, and N3 Lower border_0 are dominant features, with the top-ranked feature showing slightly higher importance than in SMOTE. Many of the top features overlap between SMOTE and ROS, suggesting a stable set of core predictors regardless of the balancing method. The ROS curve indicates a steep decline in importance after the top few features, suggesting that ROS-trained models rely more on a limited set of predictors. This may result from ROS duplicating minority samples without adding synthetic variability, leading to a focus on the strongest discriminative features.

Both methods highlight that certain anatomical measurements, particularly gonial angles and lower border profiles, are crucial for classification. The model’s robustness is enhanced by SMOTE, which distributes the importance across a wide range of features. On the other hand, ROS focuses on fewer, highly discriminative features, which may increase sensitivity to changes in those measurements.

**Fig. 10:**
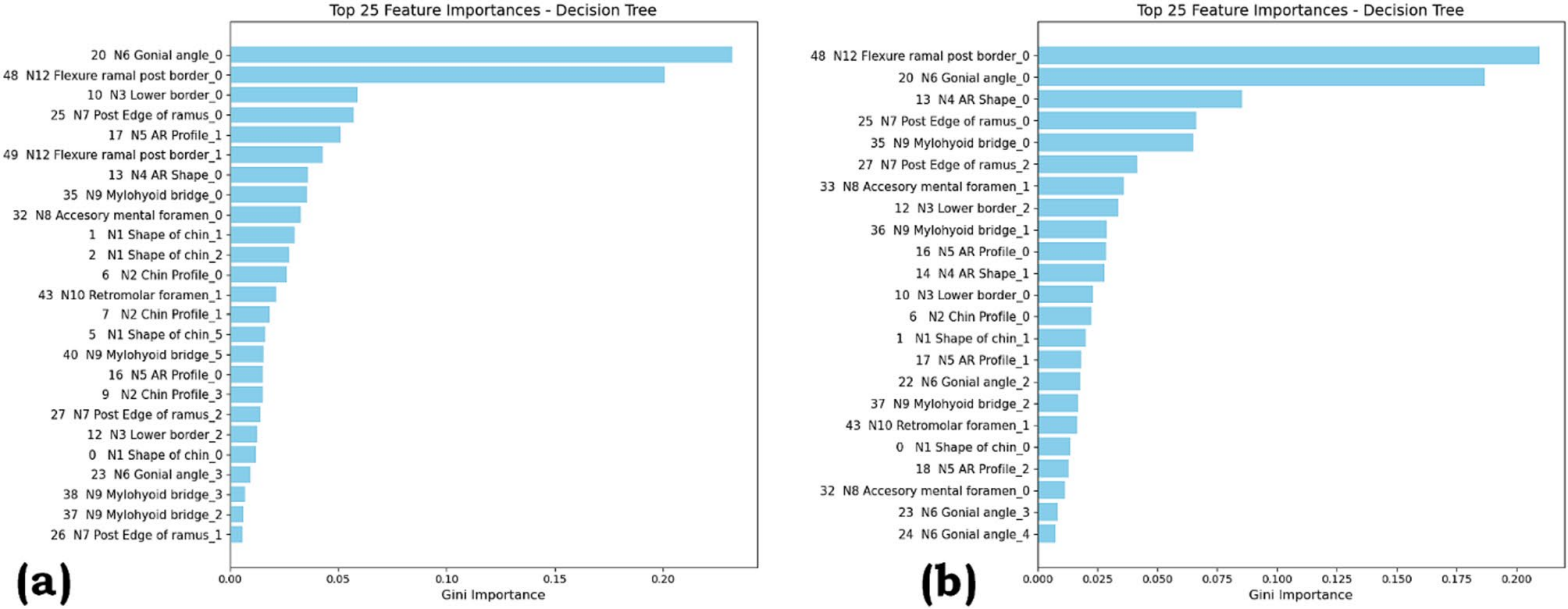
Gini index plot for decision tree with (**a**) SMOTE (**b**) ROS.

The plot for the Gini index of the decision tree is shown in Fig. 10 for SMOTE and ROS. Gini importance measures each feature’s impact on reducing node impurity, with higher values indicating a stronger role in classification.

For the SMOTE, N6 Gonial angle_0 and N12 Flexure ramal post border_0 are the dominant features, having importance scores above 0.10. Other contributors include N3 Lower border_0, N7 Post Edge of ramus_0, and N5 AR Profile_1. The importance values are relatively varied across features, suggesting that SMOTE enables the model to utilize a broader set of predictors.

For the ROS, N12 Flexure ramal post border_0 and N6 Gonial angle_0 remain the top features with higher importance, but with even higher importance, particularly for the top feature, which exceeds 0.19. The ranking also shows a slightly steeper drop after the top few features, indicating stronger reliance on key variables such as N4 AR Shape_0 and N7 Post Edge of ramus_0. The overlap in leading features between SMOTE and ROS reflects their consistent predictive value, while the sharper concentration in ROS suggests a heavier dependence on a smaller feature set.

### Model performance variability

**Fig. 11:**
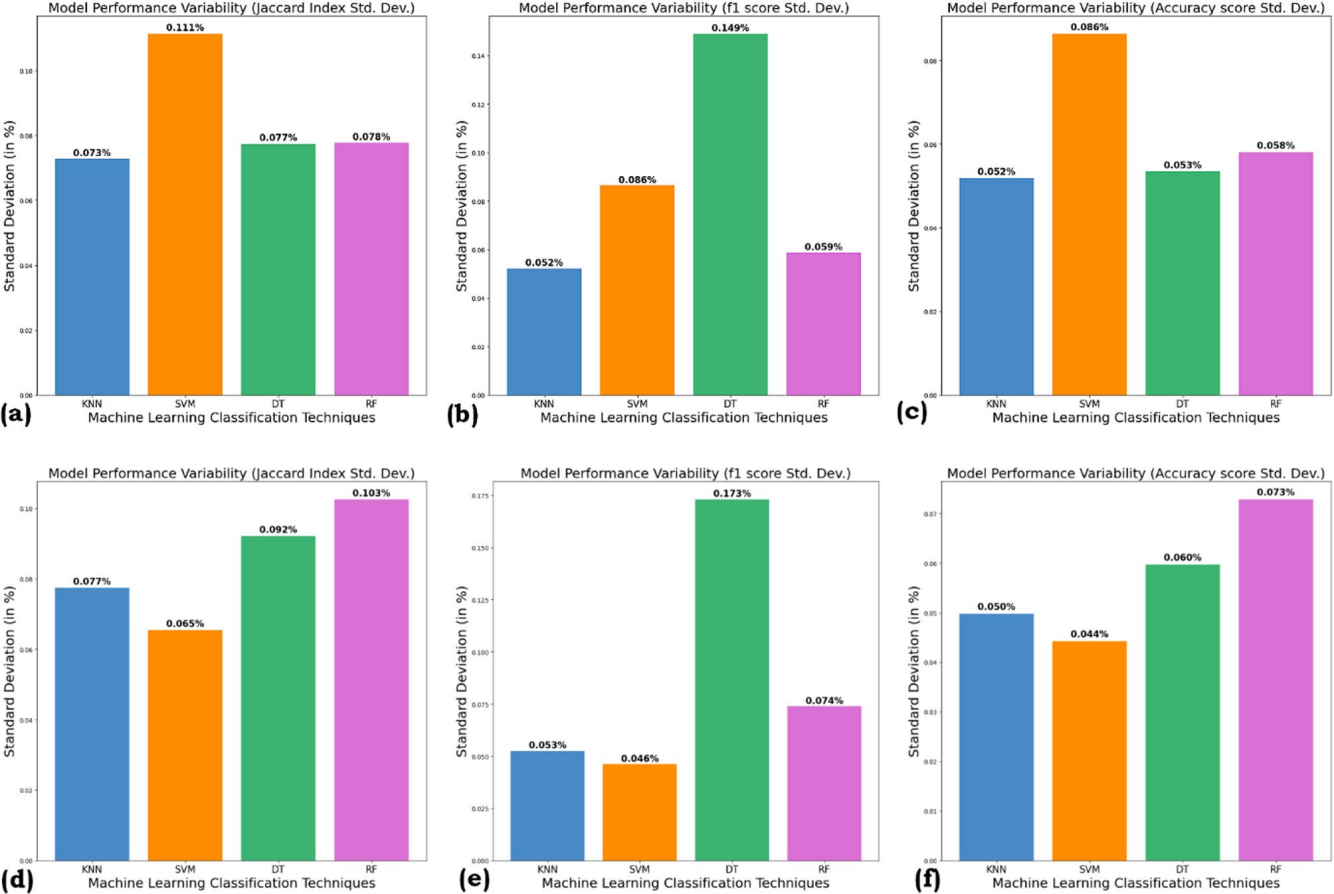
Standard deviation for (**a**) Jaccard Index with SMOTE, (**b**) F1 Score with SMOTE, (**c**) Accuracy score with SMOTE, (**d**) Jaccard Index with ROS, (**e**) F1 Score with ROS, (**f**) Accuracy Score with ROS.

The plot for the standard deviation for the Jaccard Index, F1 score, and accuracy score for all the ML algorithms in this study is presented in Fig. 11 for both SMOTE and ROS methods.

Standard deviation is used to measure performance variability, with lower values indicating more consistent results across multiple runs. For SMOTE, SVM records the highest variability for the Jaccard Index (0.111%) and Accuracy Score (0.086%), while DT shows the highest variability for the F1 Score (0.149%). The lowest variability for Jaccard Index, F1 score, and accuracy for KNN is 0.073%,0.052% and 0.052% respectively, for KNN, which depicts its strong stability. RF and DT show moderate variability, with RF performing better than DT for F1 Score stability but slightly worse for Jaccard Index consistency.

For ROS, RF exhibits the highest variability for Jaccard Index (0.103%) and Accuracy Score (0.073%), while DT again shows the largest fluctuation for the F1 Score (0.173%). The most stable model recorded was KNN, having the lowest variability score for F1 (0.035%) and Accuracy score (0.05%), whereas SVM records the lowest Jaccard Index variability (0.065%).

Overall, KNN exhibits the least sensitivity to variations in training data from resampling methods, performing consistently with both SMOTE and ROS. Conversely, DT and RF are are more affected by small perturbations during oversampling, particularly in the F1 Score, due to their inherent instability. SVMs, on the other hand, exhibit moderate variability but can spike with certain metrics using SMOTE, likely due to changes in support vectors from synthetic samples. Thus, simpler algorithms like KNN are more robust to resampling variability, while more complex models may require additional tuning for stability.

### SHAP waterfall and SHAP summary visualization

SHapley Additive exPlanations (SHAP) is a method by which the interpretations of the predictions of machine learning models are made. A SHAP waterfall plot visualizes an individual prediction by showing how each feature’s SHAP value contributes from the baseline (average prediction) to the final output. It reveals which features push the prediction higher or lower, offering detailed interpretability.

**Fig. 12:**
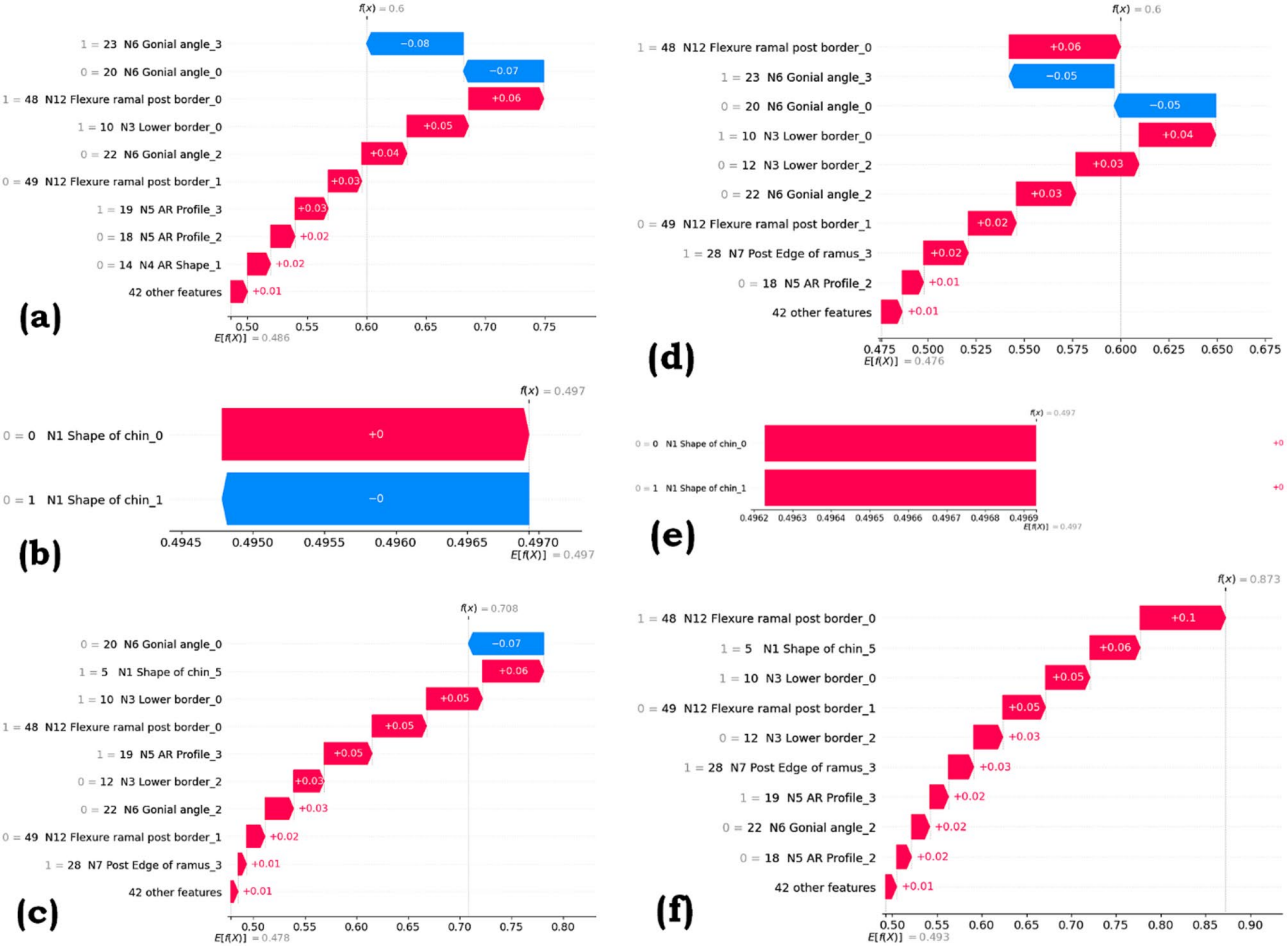
SHAP Waterfall plot for (**a**) KNN with SMOTE, (**b**) DT with SMOTE, (**c**) SVM with SMOTE, (**d**) KNN with ROS, (**e**) DT with ROS, (**f**) SVM with ROS.

**Fig. 13:**
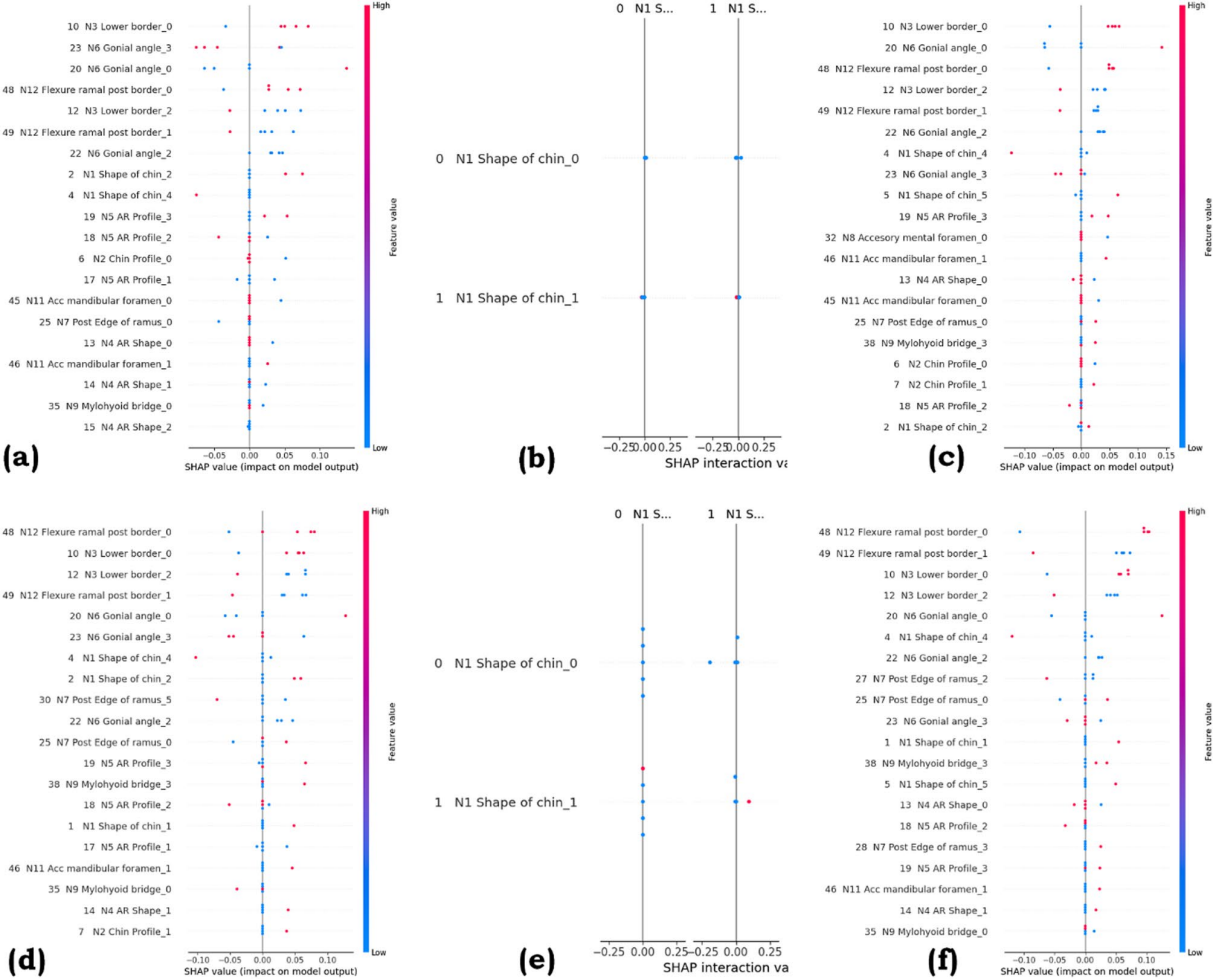
SHAP Summary plot for (**a**) KNN with SMOTE, (**b**) DT with SMOTE, (c) SVM with SMOTE, (d) KNN with ROS, (e) DT with ROS, (f) SVM with ROS.

In contrast, a SHAP summary plot provides a global view of feature importance across the dataset, illustrating the magnitude and direction of each feature’s impact. This plot combines feature importance with SHAP value distribution, helping us identify the most significant features and their influence on predictions. The Figs. 12 and 13 show the SHAP waterfall and summary plots for KNN, DT, and SVM for SMOTE and ROS, respectively.

The SHAP waterfall plots in Fig. 12 indicate how individual features contribute to specific model predictions for different algorithms and resampling techniques. In each plot, the baseline prediction E[f(X)] is adjusted in a stepwise manner by the features that influence the most, with red bars indicating features that push the prediction higher and blue bars showing those that push it lower. In the KNN model with SMOTE (a) and Random Over-Sampling (ROS) (d), features like N6 Gonial angle_3 and N12 Flexure ramal post border_0 show significant effects. In the Support Vector Machine (SVM) models (c, f), N12 Flexure ramal post border_0 has a strong positive influence. Decision Tree models (b, e) are simpler, often dominated by a single feature like N1 Shape of chin, indicating lower complexity and more discrete decision rules.

Figure 13 presents SHAP summary plots, which display the overall importance and direction of influence of each feature across all predictions for different models and resampling strategies. In KNN and SVM models with both SMOTE and ROS (a, c, d, f), features such as N3 Lower border_0, N6 Gonial angle, and N12 Flexure ramal post border emerge as consistently high-impact predictors, with their SHAP values indicating whether higher feature values push predictions toward positive or negative outcomes. In contrast, Decision Tree models (b, e) show a more simplified influence landscape, often dominated by a single categorical feature like N1 Shape of chin, resulting in minimal variation in SHAP values across instances. The color gradient reflects the feature value (red for high, blue for low), while the horizontal spread indicates the strength and variability of each feature’s contribution to the model output.

## Discussion

This study observed 12 mandibular non-metric parameters in a sample of 156 individuals, consisting of 102 males and 54 females. Forensic and physical anthropology primarily focus on sexual dimorphism, the observable differences between the male and female skeletal features^38^. Sex determination is greatly influenced by the morphological and morphometric characteristics of the human mandible^39^. Studies have shown that the chin profile, gonial flares, and prominent muscle markings are some of the important features used in sex determination using nonmetric parameters^40^. Machine learning algorithms are successfully used in medical applications, particularly in sex determination using human anthropometric data. E. C. Küchler et al.,^41^ conducted a study using mandibular and dental dimensions for sex determination and concluded that the machine learning algorithms found these dimensions useful for sex identification. Epain et al.,^42^ used coxal bones and CT scans of 580 living individuals for sex determination. The study employed the ANN machine learning algorithm, achieving an accuracy of over 97%. Savall et al.,^43^ utilized metric data from coxal bones with 11 distinct measurements for sex determination. A sample size of 113 individuals was used, and a decision tree machine learning algorithm was applied with a tenfold cross-validation, achieving an accuracy of 92%. Machine learning models like SVM was used to determine sex and age based on the teeth X-ray images^44^.

Research conducted by Hu et al.,^31^ examined 107 Korean mandibles, consisting of 74 male and 33 female specimens, focusing on 13 nonmetric characteristics. The results indicated that most male mandibles (68.1%) displayed a rocker shape, while most female mandibles (84.6%) were straight. Current analysis of 156 mandibles (102 male and 54 female), 70.6% of the male mandibles exhibited a rocker-shaped inferior border, while 74.1% of the female mandibles displayed a straight lower border.

It was observed that the posterior margin of the ramus was predominantly curved in both males (69.3%) and females (69.2%), which Hu et al.,^31^ noted as being unreliable for sex determination. Loth and Henneberg conducted a nonmetric study on the curvature degree of the posterior ramus border among Africans and found significant differences between the sexes, achieving 99% accuracy in identification. Similarly, Indrayana et al.,^45^ determined that male and female Indonesians could be distinguished with 90% and 94% accuracy, respectively. Coqueugniot et al.,^46^ and Saini et al.,^47^ concluded that ramus flexure is an effective characteristic for sex determination, with average accuracy rates of up to 82%. However, in the current study, 76.5% of males had curved posterior borders, while 63% of females had straight borders, indicating that flexure is predominantly a male trait.

Notably, 91.7% of males exhibited either a bilobate or square chin, whereas 54.5% of females had a pointed chin, according to Hu et al.,^31^. In the current study, chin shapes and profiles were similarly distributed among males and females, with no significant statistical differences. This finding suggests that chin shape alone is not a reliable characteristic for sex differentiation. Furthermore, shapes and profiles of the ascending ramus, and the presence of accessory foramina, did not show significant variation between male and female mandibles.

This work employed the four most used classification algorithms for sex determination on 156 dry mandibles using nonmetric parameters. The ML algorithms were evaluated using the metrics of the Jaccard index and F1 scores. The Jaccard Index and F1 scores for the four ML algorithms under both SMOTE and ROS oversampling. KNN recorded the lowest performance, with SMOTE scores of 0.67 (Jaccard) and 0.80 (F1), and ROS scores of 0.78 and 0.87, indicating sensitivity to noisy and imbalanced data. Decision Tree (DT) outperformed KNN, achieving up to 0.82 (Jaccard) and 0.90 (F1) under ROS, reflecting its ability to form effective hierarchical decision boundaries for non-linear data. SVM produced results similar to DT in Jaccard scores but slightly lower F1 scores, suggesting higher precision but reduced recall. Random Forest (RF) delivered the best results overall, with the highest Jaccard (0.86) and F1 (0.92) scores, attributed to its ensemble structure that reduces overfitting and captures complex feature interactions.

To enhance the external validity of a study on South Indian mandibles and sexual dimorphism, it is vital to ensure the findings are applicable beyond the original sample. This can be achieved by incorporating data from diverse regional and ethnic groups across India and internationally. Including specimens of various ages and backgrounds will account for variability due to nutrition and lifestyle. Standardizing observation protocols and employing validated scoring methods will improve reproducibility. Collaborating with multiple institutions for larger, geographically diverse samples will reduce bias and strengthen the relevance of the results for forensic, anthropological, and clinical applications^48,49^.

For the accuracy and confidence intervals, RF again led with 0.92 for both SMOTE and ROS, outperforming other algorithms by 8–15%. DT and SVM improved from ~ 0.82 under SMOTE to 0.90 under ROS, while KNN increased from 0.80 to 0.87. Table 5 presents specificity per class, with RF achieving the highest baseline for both Males (0.86) and Females (1.00), consistent across oversampling methods. ROS generally improved Male specificity for most algorithms, with KNN, DT, and SVM all showing gains. McNemar’s test results revealed no statistically significant accuracy differences between any algorithm pairs for either SMOTE or ROS, with all p-values above 0.05. This indicates that while RF consistently yields the highest metrics, differences in accuracy across models are not statistically significant. Overall, RF demonstrates the most robust and balanced performance, maintaining high accuracy, specificity, and F1 scores regardless of the oversampling technique, while ROS often offers modest improvements for other models.

Bootstrap AUROC difference analysis showed that for SMOTE, differences ranged from − 0.15 to 0.12, and for ROS from − 0.04 to 0.06, with all 95% confidence intervals including zero. This indicates no statistically significant AUROC differences between model pairs under either resampling method. Overall, all models exhibit comparable classification accuracy regardless of the oversampling technique used.

Balanced accuracy results show that ROS improves performance for KNN, DT, and SVM compared to SMOTE, with increases of 2.5%, 9.7%, and 5.8% respectively, while RF remains unchanged at 0.92, indicating robustness to the oversampling method.

Matthews Correlation Coefficient results also indicate that ROS generally outperforms SMOTE, with notable gains for KNN (0.61→0.78), SVM (0.65→0.80), and DT (0.72→0.80).

Random Forest achieves the highest MCC (0.86) and shows consistent performance across both balancing methods.Overall, ROS provides a slight advantage for most algorithms, while RF delivers stable, high performance regardless of the method used.

Permutation Feature Importance and Gini Index compared the top 25 features for Random Forest (permutation importance) and Decision Tree (Gini importance) under SMOTE and ROS. Across both models, features such as N6 Gonial angle and N12 Flexure ramal post border consistently rank highest, confirming their strong predictive value. SMOTE produces a flatter importance distribution, indicating broader feature utilization, while ROS shows a steeper drop after the top few features, reflecting heavier reliance on a smaller subset. These results suggest SMOTE promotes model robustness through feature diversity, whereas ROS concentrates predictive power but may increase sensitivity to variations in dominant predictors.

The SHAP waterfall plots show how individual features adjust specific model predictions from the baseline, with red bars increasing and blue bars decreasing the output. KNN and SVM models highlight features like N6 Gonial angle and N12 Flexure ramal post border as major contributors, while Decision Trees are often dominated by a single feature, such as N1 Shape of chin, reflecting simpler decision structures. The SHAP summary plots provide an overall view of feature importance, showing N3 Lower border, N6 Gonial angle, and N12 Flexure ramal post border as consistently influential in KNN and SVM, whereas Decision Trees again show single-feature dominance and lower variability in contributions. Color indicates feature value magnitude, and horizontal spread reflects impact strength.

## Limitations

The study had a few limitations to note. It is worth noting that smaller samples were collected to observe nonmetric parameters in the present study. Another noteworthy point is an imbalance between the available male (102) and female (54) samples, which was further evaluated with oversampling techniques like the SMOTE and ROS. Further, another thing worth noting is that the samples were collected from only one geographical location, like the South Indian population. Therefore, our further research aims to overcome these limitations by collecting larger sample sizes and collecting data from various geographical locations, thereby observing the difference in results.

## Conclusion

The present study used the most commonly used classification algorithms like KNN, DT, SVM, and RF. Based on the evaluation metrics and model comparison, RF consistently demonstrated superior performance across all key indicators. It achieved the highest scores in both Jaccard Index and F1 Score, confirming its accuracy and strong alignment with ground truth labels.

The comparative evaluation of four machine learning algorithms—K-Nearest Neighbors (KNN), Decision Tree (DT), Support Vector Machine (SVM), and Random Forest (RF)—under two oversampling methods, SMOTE and Random Over-Sampling (ROS), reveals distinct performance patterns. Random Forest consistently outperformed all other models across multiple metrics including Jaccard Index, F1 score, accuracy, specificity, and MCC, demonstrating its robustness and ability to capture complex feature interactions while reducing overfitting. Both Decision Tree and SVM showed competitive results, with ROS generally improving their performance more noticeably than SMOTE. KNN, while showing sensitivity to noisy and imbalanced data, benefited from oversampling, especially with ROS, but still lagged behind the ensemble and margin-based models in overall accuracy and robustness.

The statistical tests, including McNemar’s test and bootstrap AUROC difference analysis, confirm that although Random Forest yields the highest performance scores, the differences in accuracy and AUROC among the models were not statistically significant under either oversampling approach. This indicates that all models provide comparable classification capability in the context of the dataset used. However, the balanced accuracy and MCC metrics suggest that ROS tends to offer slight advantages for most algorithms by improving sensitivity and predictive consistency, except for Random Forest which remained stable and reliable regardless of oversampling method. These findings highlight the practical value of ROS in enhancing model generalization for less complex classifiers while reaffirming the superior stability of Random Forest in handling imbalanced data.

Feature importance analyses through permutation importance, Gini index, and SHAP value visualizations consistently emphasize the critical predictive role of features such as the N6 Gonial angle and N12 Flexure ramal post border across models and sampling methods. SMOTE encourages a more distributed reliance on a wider set of features, potentially fostering robustness through diversity, whereas ROS focuses the model’s predictive power on a narrower set of dominant features, which might increase sensitivity to feature variation. Additionally, simpler models like Decision Trees tend to rely heavily on a single key feature, limiting variability in their decision logic. Overall, the interpretability insights support the quantitative results, confirming that Random Forest’s ensemble approach not only boosts performance but also balances feature utilization effectively, making it the most suitable model for this classification task.

In conclusion, among the evaluated algorithms, Random Forest is the best-performing and most reliable model. It consistently achieves the highest accuracy, specificity, and robustness across different oversampling methods, demonstrating resilience to data imbalance and noise. While Decision Tree and SVM offer reasonable alternatives and benefit from oversampling, they do not match Random Forest’s overall predictive power and stability. KNN, though improved by oversampling, remains less effective for this task. Therefore, Random Forest is recommended as the optimal model for achieving strong, consistent classification performance in this context.

## Data Availability

The data generated in this research is publicly available on Figshare. The link is provided: [https://figshare.com/s/7b6970ef73e43e78ea97](https:/figshare.com/s/7b6970ef73e43e78ea97)DOI: 10.6084/m9.figshare.28551953.

## Abbreviations

AUC: Area under the curve
FN: False negatives
FP: False positives
KNN: k-nearest neighbors
CBCT: Cone beam computed tomography
DT: Decision tree
RF: Random forest
SVM: Support vector machines
LR: Logistic regression
MCC: Matthews correlation coefficient
ML: Machine learning
SMOTE: Synthetic minority oversampling technique
ROC: Receiver operating characteristic
ROS: Random oversampling
TP: True positives
