# Correction: Estimation of sexual dimorphism of adult human mandibles of South Indian origin using non-metric parameters and machine learning classification algorithms

**DOI:** 10.1038/s41598-026-44683-2

**Published:** 2026-03-23

**Authors:** John Valerian Corda, A. Karthikeyan, Mohammad Zuber, Meera Jacob, Amith Ramos, Mamatha Hosapatna, Anne Dsouza, Akhilesh Kumar Pandey, Vrinda Hari Ankolekar

**Affiliations:** 1 Department of Mechanical Engineering, Moodlakatte Institute of Technology, Kundapura, Karnataka India; 2 Department of Computer Science & Engineering (Data Science), Moodlakatte Institute of Technology, Kundapura, Karnataka India; 3 Department of Aeronautical & Automobile Engineering, Manipal Institute of Technology, Manipal Academy of Higher Education, Manipal, 576104 India; 4 Department of Anatomy, Yenepoya Medical College, Mangaluru, India; 5 Department of Anatomy & Medical Imaging, American University of Antigua College of Medicine, Antigua, Antigua and Barbuda; 6 Department of Anatomy, Kasturba Medical College, Manipal Academy of Higher Education, Manipal, 576104 India; 7 Department of Community Medicine, Kasturba Medical College, Manipal Academy of Higher Education, Manipal, 576104 India

Correction to: *Scientific Reports* 10.1038/s41598-025-17831-3, published online 03 October 2025

Mamatha Hosapatna and Anne Dsouza were omitted as a corresponding author in the original version of this Article. Correspondence and requests for materials should also be addressed to mamatha.h@manipal.edu and anne.dsouza@manipal.edu.

The original Article has been corrected.

